# Adipocyte Hypertrophy, Inflammation and Fibrosis Characterize Subcutaneous Adipose Tissue of Healthy, Non-Obese Subjects Predisposed to Type 2 Diabetes

**DOI:** 10.1371/journal.pone.0105262

**Published:** 2014-08-22

**Authors:** A. M. Josefin Henninger, Björn Eliasson, Lachmi E. Jenndahl, Ann Hammarstedt

**Affiliations:** The Lundberg Laboratory for Diabetes Research, Department of Molecular and Clinical Medicine, the Sahlgrenska Academy at the University of Gothenburg, Gothenburg, Sweden; The Ohio State University, United States of America

## Abstract

**Background:**

The adipose tissue is important for development of insulin resistance and type 2 diabetes and adipose tissue dysfunction has been proposed as an underlying cause. In the present study we investigated presence of adipocyte hypertrophy, and gene expression pattern of adipose tissue dysfunction in the subcutaneous adipose tissue of healthy, non-obese subjects predisposed to type 2 diabetes compared to matched control subjects with no known genetic predisposition for type 2 diabetes.

**Method:**

Seventeen healthy and non-obese subjects with known genetic predisposition for type 2 diabetes (first-degree relatives, FDRs) and 17 control subjects were recruited. The groups were matched for gender and BMI and had similar age. Glucose tolerance was determined by an oral glucose tolerance test and insulin sensitivity was calculated using HOMA-index. Blood samples were collected and subcutaneous abdominal adipose tissue biopsies obtained for gene expression analysis and adipocyte cell size measurement.

**Results:**

Our findings show that, in spite of similar age, BMI and percent body fat, FDRs displayed adipocyte hypertrophy, as well as higher waist/hip ratio, fasting insulin levels, HOMA-IR and serum triglycerides. Adipocyte hypertrophy in the FDR group, but not among controls, was associated with measures of impaired insulin sensitivity. The adipocyte hypertrophy was accompanied by increased inflammation and Wnt-signal activation. In addition, signs of tissue remodeling and fibrosis were observed indicating presence of early alterations associated with adipose tissue dysfunction in the FDRs.

**Conclusion:**

Genetic predisposition for type 2 diabetes is associated with impaired insulin sensitivity, adipocyte hypertrophy and other markers of adipose tissue dysfunction. A dysregulated subcutaneous adipose tissue may be a major susceptibility factor for later development of type 2 diabetes.

## Introduction

Obesity is a major driving force promoting the diabetes epidemic and the expanding adipose tissue plays a crucial role for obesity-associated insulin resistance [1] and is considered to have a central role in metabolic regulation [2].

Adipose tissue expansion occurs mainly through two processes; expansion of existing adipocytes (hypertrophy) and/or recruitment of new adipocytes (hyperplasia). Hypertrophic, rather than hyperplastic, obesity has long been known to be related to insulin resistance and other aspects of the metabolic syndrome [3]–[5] and to be an independent predictor for future type 2 diabetes [6], [7].

Numerous studies have demonstrated that adipose tissue dysfunction contributes to unfavorable metabolic changes and type 2 diabetes. Key characteristics of a dysfunctional adipose tissue are, in addition to enlarged adipose cells, impaired adipocyte differentiation, inflammation, remodeling and fibrosis [8], [9] and it has been shown to be related to impaired commitment and differentiation of adipocyte precursor cells [10], [11].

Adipocyte differentiation is a complex process tightly regulated by transcriptional regulators whose induction are necessary for adipogenesis and insulin sensitivity [12], but also by the Wnt-signaling pathway whose inhibition is a prerequisite for preadipocyte differentiation [13], [14]. Inappropriate alteration of these pathways is well known to be associated with obesity-related metabolic complications [15]–[17].

It has also long been known that obesity is associated with a low-grade chronic inflammation residing in the adipose tissue. In 2003 two noteworthy publications [18], [19] demonstrated the involvement of adipose tissue macrophage infiltration in obesity and insulin sensitivity, since then the importance of these findings for the development of insulin resistance and type 2 diabetes has been under investigation [20].

There is also a clear connection between adipocytes and macrophages in terms of adipose tissue expansion-related remodeling and its relation to insulin resistance. The remodeling process its associated with local hypoxia, adipocyte cell death and enhanced chemokine secretion dependent on macrophages to create a permissive environment. Adipocytes, in turn, are responsible for the initiation of the macrophage infiltration [9].

However, the chronic inflammatory and hypoxic milieu also activates tissue fibrosis, which becomes pathogenic when not tightly regulated, resulting in changes of the normal tissue structure and function. Recently, increased human adipose tissue fibrosis has been associated with obesity and insulin resistance [21], [22].

Most of the findings associating different aspects of adipose tissue dysfunction with type 2 diabetes have been demonstrated in subjects already affected by obesity and/or type 2 diabetes. In the present study, we investigated the presence of adipocyte hypertrophy and alteration of genes involved in adipose tissue dysfunction in subcutaneous adipose tissue from non-obese, glucose tolerant subjects with known genetic predisposition for type 2 diabetes compared to matched control subjects without known genetic predisposition.

Our findings show that in spite of similar age, BMI and percent body fat, FDRs displayed adipocyte hypertrophy that was associated with impaired insulin sensitivity and other early signs of adipose tissue dysfunction.

## Materials and Methods

### Subjects

Seventeen subjects with at least one known first-degree relative with type 2 diabetes (FDRs) and 17 subjects without known genetic predisposition for type 2 diabetes (controls) were recruited for this study via newspaper advertisements. All subjects were non-obese, had normal glucose tolerance, and the groups were matched for gender and BMI and had similar ages.

### Ethics statement

The studies were approved by the local Ethical Committees at the Sahlgrenska Academy at the University of Gothenburg and were performed in agreement with the Declaration of Helsinki. All subjects received written information and gave written consent to participate.

### Biochemical and anthropometric measures

Height and weight were measured to the nearest cm and 0.1 kg and BMI calculated as kg body weight divided by height (m) squared. Amount of fat and lean body mass (FM and lbm respectively) were measured by bioimpedance. Fasting blood samples were drawn after an over night fast, followed by an oral glucose tolerance test (OGTT) (75 g glucose) to evaluate glucose tolerance (blood samples were taken at 0, 30, 90 and 120 min). Circulating plasma glucose was determined using a photometric method by the accredited central hospital laboratory and insulin concentrations by a micro-particle enzyme immunoassay (Abbott Laboratories, Tokyo, Japan). Plasma lipid concentrations were determined by standard methods in the accredited central hospital laboratory. The Homeostasis Model Assessment (HOMA-IR) was used as an indirect measure of insulin sensitivity [23]. Circulating adiponectin levels were measured in serum by a human adiponectin ELISA-kit (B-Bridge International, Sunnyvale, CA, USA). Circulating IL-1b and IL-1RN were measured in serum by Quantikine ELISA (R&D systems, Minneapolis, MN, USA).

### Adipose tissue biopsies

Human abdominal subcutaneous adipose tissue was obtained in the fasting state by needle biopsy. Tissue was either directly processed for RNA extraction or treated with collagenase for adipocyte isolation. Isolation of adipocytes was performed essentially as previously described [10]. Briefly, biopsies were washed to remove traces of blood and treated with collagenase (1 mg/ml) (Sigma, St Louise, MO, USA) for ∼60 min at 37°C in a shaking waterbath. Isolated adipocytes were filtered through a 250 µm nylon mesh, washed with fresh medium. Adipocyte cells were placed on a siliconized glass slide and 100 consecutive cell diameters were measured with a calibrated ocular or processed for RNA extraction.

### RNA extraction and quality control

Total cellular RNA was extracted from abdominal subcutaneous adipose tissue and isolated adipocytes with the guanidinium thiocyanate method as described [24]. RNA quality control was performed using the Agilent RNA 6000 Nano Assay and 2100 Bioanalyzer (Agilent Technologies, Waldbronn, Germany) and concentration determined using NanoDrop 1000 (Thermo Scientific/Saveen Werner, Limhamn, Sweden).

### Gene expression analysis

Gene expression was analyzed with gene array micro fluid cards and the ABI PRISM 7900HT sequence detection system (Applied biosystems, Foster City, CA, USA). The micro fluid cards were custom made using pre-designed gene expression assays (Applied biosystems, Foster City, CA, USA) as indicated in Table S1. Each sample was run in duplicate and the quantity of a particular gene in each sample was normalized to ribosomal *18s* RNA.

### Statistical analysis

All data are presented as mean ± SD calculated with conventional methods. Data was tested for normality using the Shapiro-Wilks test. The Mann-Whitney's U test for independent observations was performed to compare differences between groups. Correlations were performed on natural log transformed data using PASWstatistics (SPSS Inc). A p-value <0.05 was considered to be statistically significant.

## Results

### Phenotypic characterization

All subject were non-obese and had normal glucose tolerance after an oral glucose challenge (75 grams). The groups were matched for BMI and gender and did not differ in age or number of nicotine users (three in each group) (Table 1).

**Table 1:** Clinical characteristics.

Variable	Control	FDRs	P-value
Gender (male/female)	6/11	6/11	
Age (years)	34±8	38±9	n.s.
BMI (kg/m2)	24.1±2.37	24.7±2.31	n.s.
WHR	0.80±0.06	0.85±0.06	0.008
FM (kg)	16.6±5.3	18.6±7.0	n.s.
FM (%)	22.9±7.4	25.3±9.1	n.s.
Adipocyte cell size (um)	88±9	96±10	0.016
fp-glucose (mmol)	4.5±0.3	4.8±0.5	0.064
fs-insulin (pmol/l)	34.3±11.6	46.4±13.8	0.003
HOMA-IR	1.00±0.40	1.44±0.45	0.02
OGTT 2 h glucose (mmol/l)	5.0±0.8	5.4±1.1	n.s.
s-triglycerides (mmol/l)	0.71±0.18	1.00±0.34	0.011
s-cholesterol (mmol/l)	4.49±0.89	4.78±0.76	n.s.
s-HDL cholesterol (mmol/l)	1.49±0.18	1.54±0.42	n.s.
s-LDL cholesterol (mmol/l)	2.52±0.62	2.87±0.63	n.s.
s-adiponectin (ug/ml)	10.5±4.6	8.3±3.4	0.098
s-IL-1b (pg/ml)	1.50±0.59	1.28±0.96	n.s.
s-IL-1RN (pg/ml)	239±64	254±126	n.s.

Data presented as Mean±SD.

FM: Fat mass.

OGTT: Oral glucose tolerance test.

WHR: Waist/hip ratio.

Measure of total body FM and calculation of percent FM (FM%) showed no difference between the groups in body composition. In spite of no differences in BMI or FM% the FDR had significantly higher WHR, a marker of abdominal obesity [3], and enlarged abdominal subcutaneous adipocytes.

The FDR had slightly elevated fasting glucose levels and significantly increased fasting insulin concentrations, compared to the controls, indicating reduced insulin sensitivity under basal conditions. Calculation of HOMA-IR confirmed a significant reduction in insulin sensitivity for the FDR compared to healthy controls.

Serum high-density lipoprotein (HDL), low-density lipoprotein (LPL) and total cholesterol levels did not differ between the groups, whereas circulating serum triglyceride levels (TG) were significantly higher in the FDRs compared to controls (Table 1).

Serum adiponectin levels did not differ significantly between the groups, although there was a trend towards reduced levels in the FDRs (Table 1).

### Adipocyte cell size and metabolic parameters

**Figure 1:**
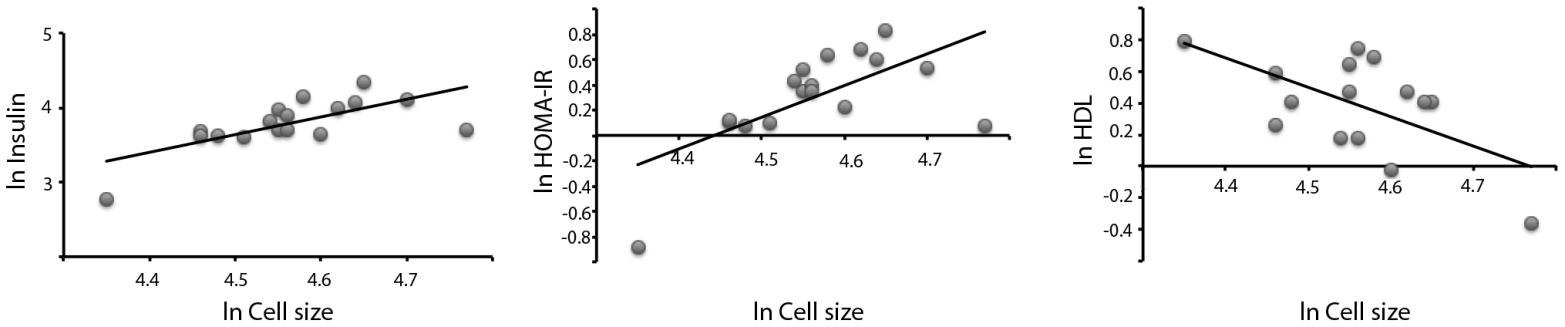
Adipocyte cell size and metabolic parameters in FDRs. Adipocyte cell size in relation to (A) fasting insulin (R = 0.69, p = 0.003), (B) HOMA-IR (R = 0.64, p = 0.006) and (C) serum HDL (R = −0.50, p = 0.019).

We examined the relation between adipocyte cell size and metabolic parameters related to impaired glucose metabolism and type 2 diabetes in the group of FDRs. Adipocyte cell size was associated with fasting insulin (R = 0.69, p = 0.003), but not glucose levels. In line with these findings, cell size was also associated with indirect measures of insulin sensitivity i.e., HOMA-IR (R = 0.64, p = 0.006) and to fasting HDL levels (R = −0.50, p = 0.019), but not to LDL or total cholesterol in the FDRs. No significant correlation between adipocyte cell size and circulating adiponectin levels could be found in this small material (Figure 1 and data not shown). Cell size did not correlate with any of the metabolic parameters measured in the matched control group probably due to the limited range of adipose cell size (data not shown).

### Wnt-signaling pathways and adipocyte differentiation

Adipose tissue differentiation and function is related to insulin sensitivity and other characteristic metabolic changes associated with type 2 diabetes. We, therefore, examined the expression level of genes known to be involved in adipocyte differentiation in adipose tissue and isolated adipocytes in the two groups.

Activation of Wnt-signaling is well-known to prevent adipocyte differentiation and therefore several target genes for this pathway such as fibronectin 1 (FN1), peroxisome proliferator-activated receptor delta (PPARD) and cyclin D2 (CCND2) were quantified, as well as the central Wnt-signaling transcription factor Transcription Factor 7-Like 2 (TCF7L2). In general, the analysis showed an increased Wnt-signaling activity in adipose tissue from FDRs compared to control subjects. The expression level of TCF7L2 was significantly upregulated, as well as the expression of CCND2. The increased expression of other Wnt-target genes did, however not, reach statistical significance.

The activity of the Wnt-signaling pathway is modulated by a number of secreted proteins. The gene expression of all regulatory proteins measured in this study, i.e., Dickkopf 1 (DKK1), DKK2, secreted Frizzled related protein 1 (SFRP1), SFRP2, WNT5a, tended to be upregulated in FDRs. However, only the upregulation of DKK2 and SFRP1 reached statistical significance (Table 2).

**Table 2:** Gene expression – Wnt-signaling.

	Adipose tissue		
Wnt-signaling	Control	FDRs	P-value
TCF7L2	0.78±0.22	1.17±0.59	0.028
FN1	0.41±0.18	0.70±0.55	n.s.
PPARD	0.70±0.17	0.92±0.50	n.s.
CCND2	0.17±0.11	0.45±0.29	0.001
DKK1	0.26±0.17	0.43±0.27	n.s.
DKK2	0.65±0.32	0.95±0.48	0.045
SFRP1	0.46±0.18	0.72±0.41	0.041
SFRP2	0.48±0.28	0.82±0.85	n.s.
WNT5A	0.49±0.27	0.49±0.34	n.s.

Data presented as Mean±SD.

To further investigate potential differences in adipocyte differentiation between controls and FDRs, we also measured gene expression of the master regulator of adipogenesis, Peroxisome proliferator activated receptor gamma (PPARG), and its co-activators PPARgamma co-activator 1 alpha (PPARGC1A) and beta (PPARGC1B), as well as three markers of the mature adipocyte phenotype; glucose transporter 4 (GLUT4/SLC24A), adiponectin (ADIPOQ) and fatty acid binding protein 4 (FABP4) in isolated adipocytes. No significant differences could be found between the groups (Table 3).

**Table 3:** Gene expression – adipogenesis.

	Isolated adipocytes		
Adipogenesis	Control	FDRs	P-value
PPARG	0.93±0.28	1.12±0.83	n.s.
PPARGC1A	0.86±0.20	1.09±0.81	n.s.
PPARGC1B	0.82±0.28	0.83±0.33	n.s.
SLC24A	1.18±0.45	1.16±0.62	n.s.
ADIPOQ	1.08±0.21	1.31±0.68	n.s.
FABP4	1.03±0.25	1.26±0.52	n.s.

Data presented as Mean±SD.

### Inflammation and remodeling in adipose tissue

It is well known that type 2 diabetes is characterized by a low-grade inflammation that largely resides in the adipose tissue. In recent years, a concept of adipose tissue remodeling in association with hypertrophic obesity, inflammation and insulin sensitivity has also been introduced. In this study, we therefore investigated potential differences in expression of genes known to be involved in inflammation and tissue remodeling between the two groups.

The majority of investigated cytokines involved in inflammation, such as Interleukin 1 beta (IL1B), IL1 receptor antagonist (IL1RN) and IL10 showed a significantly increased expression in both isolated adipocytes and adipose tissue of the FDRs. The expression of IL6 was also upregulated, but this was only significant in isolated adipocytes (Table 4).

**Table 4:** Gene expression – inflammation.

	Isolated adipocytes			Adipose tissue		
Inflammation	Control	FDRs	P-value	Control	FDRs	P-value
TLR4	0.60±0.23	0.73±0.38	n.s.	0.57±0.25	0.78±0.37	n.s.
IL1B	0.38±0.22	1.14±0.99	0.003	0.19±0.24	1.53±1.80	0.005
IL1RN	0.13±0.08	0.62±0.58	<0.001	0.09±0.06	0.34±0.22	<0.001
IL6	0.52±0.44	1.41±1.56	0.02	1.02±0.38	1.54±1.01	n.s.
IL10	0.13±0.21	0.44±0.50	0.001	0.10±0.06	0.34±0.26	<0.001
IL13	1314±6921	1887±6797	n.s.	0.55±0.53	0.60±0.36	n.s.
CCL2	0.41±0.29	0.81±0.93	n.s.	0.31±0.44	0.57±0.40	0.007
TNFA	n.d.	n.d.	-	0.15±0.13	1.01±1.06	<0.001
CD68	n.d.	n.d.	-	0.29±0.11	0.54±0.29	0.003

Data presented as Mean±SD.

n.d.: not determined.

In addition, Tumor Necrosis Factor alpha (TNFA) was highly up-regulated in adipose tissue of FDRs, as was the expression of two markers of macrophage infiltration, CD68 and monocyte chemotactic protein-1 (MCP1/CCL2), which was doubled and highly significant (Table 4).

To investigate whether the increased adipose tissue inflammation indicated by gene expression levels was reflected systemically, we measured serum IL-1b and IL-1RN levels. No significant differences could be found between the groups and neither cytokine correlated with HOMA-IR or adipocyte cell size (Table 1 and data not shown).

To investigate if adipose tissue fibrosis, in addition to adipocyte hypertrophy and inflammation, is increased in FDRs we measured the gene expression of connective tissue growth factor (CTGF) and actin alpha 2 (ACTA2), two markers of tissue fibrosis. CTGF expression was significantly upregulated in both adipose tissue and isolated adipocytes from FDRs compared to control, while ACTA2 was significantly increased in isolated adipocytes. In line with these results MMP2, an important extracellular matrix remodeling enzyme, known to be expressed and secreted from adipocytes and involved in tissue fibrosis was also significantly upregulated in isolated adipocytes from FDRs (Table 5).

**Table 5:** Gene expression – remodeling.

	Isolated adipocytes			Adipose tissue		
Remodeling	Control	FDRs	P-value	Control	FDRs	P-value
CTGF	1.40±0.94	2.90±2.52	0.01	1.57±0.79	2.82±1.65	0.038
ACTA2	0.76±0.31	1.31±0.81	0.007	1.29±0.24	1.52±0.42	n.s.
MMP2	0.33±0.37	0.56±0.51	0.008	0.48±0.21	0.70±0.39	n.s.
HIF1A	0.53±0.16	0.69±0.29	n.s.	0.81±0.27	1.03±0.47	n.s.
NOS2	0.82±0.63	1.10±0.78	n.s.	1.44±0.85	2.35±2.82	n.s.

Data presented as Mean±SD.

Adipose tissue hypertrophy is suggested to create local areas of hypoxia. However, in the present study we were not able to detect any differences in gene expression of the master regulator of hypoxia and oxygen homeostasis, hypoxia inducible factor-1 alpha (HIF1A) or its target gene nitric oxide synthase 2 (NOS2) between the two groups, although there was a significant difference in adipocyte cells size (Table 5).

## Discussion

Obesity is a major driver of type 2 diabetes. However, obesity *per se* does not necessarily lead to type 2 diabetes, but rather individual differences in body composition, fat distribution and adipose tissue function. Numerous studies have established adipose tissue dysfunction as a contributor to metabolic imbalance and type 2 diabetes.

Most findings demonstrating the association between adipose tissue dysfunction and type 2 diabetes have been shown in mouse models or subjects with manifest obesity and/or impaired insulin sensitivity. In the present study, we investigated potential signs of dysfunctional adipose tissue in subcutaneous adipose tissue from non-obese and glucose-tolerant subjects with known genetic predisposition for type 2 diabetes compared to matched control subjects.

Subjects with genetic predisposition for type 2 diabetes constitute a well-known high-risk group for type 2 diabetes. Alterations identified in the absence of obesity and pronounced insulin resistance in these subjects can therefore potentially be considered as early changes related to later development of type 2 diabetes.

We have previously shown that genetic predisposition for type 2 diabetes, but not obesity, is associated with inappropriate expansion of the adipose cells for small increases in body fat and that this is associated with reduced insulin sensitivity [11]. In line with these findings, the results of the present study show that although the groups were matched for BMI and, importantly, in spite of no differences in FM and %FM, the adipose tissue of FDRs is characterized by cellular hypertrophy compared to matched control subjects without family history of type 2 diabetes.

A previous publication from our laboratory has shown that increased adipocyte cell size in associated with reduced number of precursor cells able to differentiate into adipocytes [10]. The finding of adipocyte hypertrophy in FDRs compared control subjects with similar BMI and %FM adds weight to the hypothesis that FDRs assume an “obese phenotype” before the conventional definition of obesity due to impaired ability to recruit and/or differentiate new pre-adipocytes. The adipocyte hypertrophy was associated with measures of impaired insulin sensitivity, suggesting that the adverse effects of adipocyte hypertrophy could be reflected within these associations.

Not only adipocyte cell size is important for adipose tissue function-related traits. Adipose tissue is not a uniform tissue and different depots have both overlapping and distinct characteristics. A recent, elegant study performed in mice, highlights these differences by demonstrating large variability in *in vivo* adipogenic potential between various fat depots [25]. Both amount of deep subcutaneous and visceral adipose tissue correlates with an increased risk of diabetes [26], [27], while subcutaneous fat in the gluteofemoral region and the superficial depot has been shown to be more beneficial and associated with improved insulin sensitivity and reduced risk of diabetes [28]–[31]. The respective contribution of different adipose tissue depots to type 2 diabetes is subject to an ongoing discussion.

Characterizing the molecular mechanisms underlying a dysfunctional adipose tissue is essential for prevention and treatment of associated metabolic disorders. However, so far, we are only beginning to understand this mechanistic link. A dysfunctional adipose tissue has been associated with several mechanisms at the cellular and molecular level. Impaired vascularization, local hypoxia, changes of cellular and intracellular matrix composition, infiltration of immune cells and increased inflammation and apoptosis are all examples of abnormalities associated with a in dysfunctional adipose tissue (reviewed in [32]).

In the present study, we found signs of an increased inflammatory state in adipose tissue of FDRs compared to control subjects. Both CD68, which is a marker of the macrophage lineage, and MCP-1 were present at a higher expression level in the FDRs suggesting increased infiltration of immune cell. Other, well-known inflammatory markers like TNFalpha and IL1B were also upregulated as well as the immune and inflammatory response modulator IL1RN, previously shown to be associated with reduced insulin sensitivity [33].

To investigate whether if the adipose tissue inflammation was reflected systemically, we also measured circulating cytokine concentrations. Due to limited serum availability and the fact that IL-6 and MCP-1 and their relation to adipose cell size have been investigated else where [34]–[36], we investigated serum levels of IL1B and IL1RN and their potential relation to adipose cell size. In the present study, we could not find a significant difference between the two groups for either cytokine, although the trends were in line with previous publication [37]. In addition, no significant correlations with either adipocyte cell size or HOMA-IR could be found for any of the two cytokines. The lack of a significant difference between the groups and correlation with insulin sensitivity in the present study could potentially be explained by the low number of subjects included compared to previous reports [33], [37].

Macrophage infiltration and inflammation has previously been shown to increase proportionally with increased BMI, body fat and adipocyte hypertrophy [18], [38]. However, in our study we found that an elevation of inflammatory markers in the adipose tissue of lean FDRs is primarily related to adipocyte hypertrophy since there was no difference in BMI or %BF between the two groups of non-obese individuals.

Local hypoxia is suggested to be involved in initiation and progression of the inflammatory state of the adipose tissue [39]. In the present study, we could not detect any difference in the expression of the hypoxic transcription factor HIF-1alpha or its target gene NOS2. However, the exact mechanism how adipose tissue hypoxia contributes to adipose tissue dysfunction is not known and is possibly more important in the presence of obesity or ongoing adipose tissue expansion.

Interestingly, we also found expression of the M2 macrophage phenotype marker IL10 [40] to be higher in the FDRs compared to control subjects. The observation of M2 macrophages in adipose tissue has been associated with areas of fibrosis and related to obesity and insulin resistance [41]. In line with these findings, two genes involved in tissue fibrosis, CTGF and ACTA2 were also significantly upregulated in the FDRs.

The dynamics of fibrosis is regulated by MMPs, which is a protein family that cleaves collagenous proteins, enabling remodeling of the extra cellular matrix. One of the MMPs produced by the adipose tissue [42] and important for adipose tissue expansion [43], MMP2, was also upregulated in the FDRs. Thus, inflammation and tissue fibrosis and remodeling seem to go hand in hand and are associated with adipocyte hypertrophy, rather than BMI or body fat, in FDRs of type 2 diabetic patients.

Activation of the Wnt-signaling pathway is associated with adipocyte hypertrophy and insulin resistance [15]–[17], which was confirmed also in the present study. This finding is in agreement with our previous publications showing that hypertrophic obesity is associated with canonical Wnt activation in the stromal cells, inhibiting adipocyte precursor cell recruitment in the adipose tissue [16].

It is well established that impaired adipocyte differentiation is related to insulin resistance and type 2 diabetes [15], [44]. However, no differences were seen in the expression level of PPARgamma and other adipogenic genes between the groups, indicating that the preadipocytes that have been able to enter the differentiation process have differentiated well and that the mechanisms underlying adipocyte hypertrophy and adipose tissue dysfunction intervene at an earlier stage of precursor cell commitment and/or pre-adipocyte recruitment. We did, however, see a trend towards reduced circulating adiponectin levels, a marker of impaired adipose tissue function and reduced insulin sensitivity, among the FDRs. This finding is in line with previous publications by our group [45], although the difference was not significant for this small group of individuals.

Taken together, the findings of the present study show that in spite of no differences in BMI or %BF, non-obese and glucose-tolerant subjects with a genetic predisposition for type 2 diabetes display early changes of the abdominal subcutaneous adipose tissue such as adipocyte hypertrophy with associated impaired glucose metabolism and an “obese phenotype” including increased markers of inflammation, remodeling and Wnt-signaling activation compared to healthy control subjects lacking a known genetic predisposition.

The present study is limited by its small number of subjects and a relatively small range of adipocyte size. However, regardless of these limitations, the results provide important information in a high-risk cohort of first-degree relatives to type 2 diabetic patients. Thus, a family history of type 2 diabetes is associated with adipocyte dysfunction and our data suggest that this could be related to inability to recruit and differentiate new adipocytes. The potential underlying genetics for this insufficiency is currently unknown. So far, type 2 diabetes associated genes identified through the many genome wide association studies performed during recent years have only explained a small fraction of the overall heritable risk for type 2 diabetes, and primarily been related to insulin secretion [46]. Future epigenetic studies may contribute with important knowledge of the genetic regulation of mesenchymal stem cell commitment and pre-adipocyte recruitment.

## Supporting Information

Table S1 **Gene expression Assay ID.**
(DOCX)
