# Supplementary material for: Adipocyte Hypertrophy, Inflammation and Fibrosis Characterize Subcutaneous Adipose Tissue of Healthy, Non-Obese Subjects Predisposed to Type 2 Diabetes
Source: PLoS One. 2014 Aug 22;9(8):e105262. doi: 10.1371/journal.pone.0105262 (PMC4141784; doi:10.1371/journal.pone.0105262)
Supplement: Table S1 — Gene expression Assay ID. (DOCX) [file pone.0105262.s001.docx]

**Table S1.** **Gene expression Assay ID**

| **Gene name** | **Assay ID** |
| --- | --- |
| PPARG | Hs01115513 |
| PPARGC1A | Hs01016719 |
| PPARGC1B | Hs00991676 |
| SLC24A | Hs00168966 |
| ADIPOQ | Hs00605917 |
| FABP4 | Hs01086177 |
| TCF7L2 | Hs01009038 |
| FN1 | Hs01549976 |
| PPARD | Hs00987011 |
| CCND2 | Hs00153380 |
| DKK1 | Hs00183740 |
| DKK2 | Hs00997455 |
| SFRP1 | Hs00610060 |
| SFRP2 | Hs00293258 |
| WNT5A | Hs00998537 |
| TNFA | Hs00174128 |
| TLR4 | Hs01060206 |
| IL1B | Hs01555410 |
| IL1RN | Hs00893626 |
| IL6 | Hs00985639 |
| IL10 | Hs00961622 |
| IL13 | Hs00174379 |
| CD68 | Hs00154355 |
| CCL2 | Hs00234140 |
| HIF1A | Hs00936371 |
| NOS2 | Hs01075529 |
| MMP2 | Hs01548727 |
| CTGF | Hs0017014 |
| ACTA2 | Hs00426835 |
| RN18S1 | Hs03928990 |
